# Verifying the Rechargeability of Li‐CO_2_ Batteries on Working Cathodes of Ni Nanoparticles Highly Dispersed on N‐Doped Graphene

**DOI:** 10.1002/advs.201700567

**Published:** 2017-11-10

**Authors:** Zhang Zhang, Xin‐Gai Wang, Xu Zhang, Zhaojun Xie, Ya‐Nan Chen, Lipo Ma, Zhangquan Peng, Zhen Zhou

**Affiliations:** ^1^ College of Chemistry and Chemical Engineering Henan Province Key Laboratory of Utilization of Non‐Metallic Mineral in the South of Henan Xinyang Normal University Xinyang 464000 China; ^2^ School of Materials Science and Engineering National Institute for Advanced Materials Institute of New Energy Material Chemistry Collaborative Innovation Center of Chemical Science and Engineering (Tianjin) Nankai University Tianjin 300350 China; ^3^ Electroanalytical Chemistry Changchun Institute of Applied Chemistry Chinese Academy of Sciences 5625 Renmin Street Changchun 130022 China

**Keywords:** air cathodes, electrocatalysis, graphene, Li‐CO_2_ batteries, Ni nanoparticles

## Abstract

Li‐CO_2_ batteries could skillfully combine the reduction of “greenhouse effect” with energy storage systems. However, Li‐CO_2_ batteries still suffer from unsatisfactory electrochemical performances and their rechargeability is challenged. Here, it is reported that a composite of Ni nanoparticles highly dispersed on N‐doped graphene (Ni‐NG) with 3D porous structure, exhibits a superior discharge capacity of 17 625 mA h g^−1^, as the air cathode for Li‐CO_2_ batteries. The batteries with these highly efficient cathodes could sustain 100 cycles at a cutoff capacity of 1000 mA h g^−1^ with low overpotentials at the current density of 100 mA g^−1^. Particularly, the Ni‐NG cathodes allow to observe the appearance/disappearance of agglomerated Li_2_CO_3_ particles and carbon thin films directly upon discharge/charge processes. In addition, the recycle of CO_2_ is detected through in situ differential electrochemical mass spectrometry. This is a critical step to verify the electrochemical rechargeability of Li‐CO_2_ batteries. Also, first‐principles computations further prove that Ni nanoparticles are active sites for the reaction of Li and CO_2_, which could guide to design more advantageous catalysts for rechargeable Li‐CO_2_ batteries.

The pressure of reducing greenhouse gas emission and the consumption of fossil fuels have directly resulted in the increasing demand of new electrochemical energy storage systems with high energy density.^1^ CO_2_ is regarded as a leading greenhouse gas and its sustained release has been implicated in the global climate change.^2^ Various physical and chemical methods are currently under development to capture thousands of tons of CO_2_ gas emitted per year.^2^, ^3^ It is extremely attractive to utilize CO_2_ in energy storage systems;^4^, ^5^, ^6^ therefore, Li‐CO_2_ batteries have been proposed and studied in recent years.^7^, ^8^, ^9^, ^10^, ^11^

In a typical Li‐CO_2_ battery, the electrochemical reaction is demonstrated as 4Li + 3CO_2_ ↔ 2Li_2_CO_3_ + C.^7^, ^8^, ^9^, ^10^ However, the formation of the discharge product C was only observed by using porous gold or platinum net cathodes; therefore, it is essential to observe the discharge products in working air cathodes, because different cathodes especially noble metals may result in different mechanisms.^7^, ^8^, ^9^, ^12^ A recent report has suggested that superoxide radicals and dissolved oxygen generated during the charging process with Li_2_CO_3_ lead to electrolyte decomposition, rather than the direct self‐decomposition of Li_2_CO_3_ or the reaction between Li_2_CO_3_ and carbon.^13^, ^14^ However, the pre‐filled Li_2_CO_3_ and conductive carbon in the above work are rather different from the in situ newly formed Li_2_CO_3_ and C, which can be verified from the extremely high charge voltage in the former case. In addition, superoxide radicals not only have great influence on the electrolyte but also corrode the carbon specimens easily.^15^, ^16^ Also, it would benefit for understanding the reversibility of Li‐CO_2_ batteries if the CO_2_ consumption and evolution were confirmed by in situ measurements during discharge and charge processes.

Up to date, only carbon,^7^, ^8^, ^9^, ^10^, ^17^, ^18^, ^19^ Ru/C,^20^, ^21^ and Mo‐based catalysts^12^, ^22^ have been utilized in Li‐CO_2_ batteries, and relatively decent electrochemical performances were obtained. In a previous report, NiO proved effective in the decomposition of Li_2_CO_3_ reported by Li group.^23^ Here, we prepared a composite of Ni nanoparticles highly dispersed on N‐doped graphene (Ni‐NG) via hydrothermal and post‐annealing treatment, which endows Li‐CO_2_ batteries with excellent electrochemical performances. Especially, the appearance and disappearance of agglomerated Li_2_CO_3_ particles and carbon thin films were observed directly in the air cathodes upon the discharge/charge process. Compared with previous results with cathodes prefilled with Li_2_CO_3_ and carbon, our results shed new light on the rechargeability of Li‐CO_2_ batteries.

The morphology and microstructure of the as‐prepared Ni‐NG composite are presented in **Figure** **1**, and the characterization of graphene is shown in Figure S1 (Supporting Information). Figure 1a shows typical wrinkled and folded structures of Ni‐NG with porous character, and Ni nanoaparticles are uniformly dispersed on graphene (Figure 1b). The detailed structure and morphology can be further determined by high‐resolution transmission electron microscope (HRTEM). In addition to three bright rings of graphene, the selected area electron diffraction (SAED) pattern of Ni‐NG displays several diffraction rings, which can be attributed to the lattice fringes of the (111) and (220) planes of Ni nanoparticles, corresponding to the *d*‐spacing of 2.04 and 1.77 Å (Figure 1c,d), respectively. To get a clearer view of the Ni granule dispersion, characterizations were extended to the high angle annular dark field (HAADF, Figure 1e), and small white particles are obviously loaded on graphene through an “anchored model.” The element mapping of various elements in Figure 1f–i shows the uniform distribution of N, Ni, and C in Ni‐NG. The distribution of Ni and N in a large area of scanning electron microscope (SEM) is shown in Figure S2 (Supporting Information). The Ni content of Ni‐NG is estimated to be ≈27.2 wt% through thermogravimetric‐differential thermal analysis (TG‐DTA) (Figure S3, Supporting Information). More characterizations for graphene and Ni‐NG are available in Figures S4 and S5 and Table S1 (Supporting Information).

**Figure 1:**
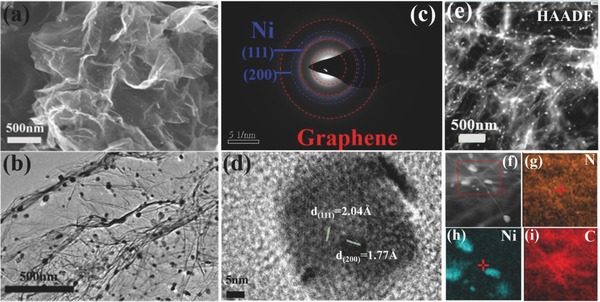
a) SEM image, b) TEM image, and c) SAED pattern of Ni‐NG. d) HRTEM image of Ni particles in Ni‐NG. e) Dark‐field TEM image of Ni‐NG. f–i) HRTEM EDS mapping of N, Ni, and C in the selected area.

As disclosed in the cyclic voltammetry (CV) curves (**Figure** **2**a), Ni‐NG exhibits more apparent cathodic and anodic peaks with higher peak current, which indicates higher catalytic activity for Li‐CO_2_ batteries, compared with the individual graphene. The discharge–charge curves are shown in Figure 2b for Li‐CO_2_ batteries with Ni‐NG cathodes at a current density of 100 mA g^−1^, and the Ni‐NG cathode delivers a capacity of 17 625 mA h g^−1^, which is 2.7 times that with the graphene cathode,^7^ and much higher than those of other carbon cathodes.^8^, ^9^, ^10^ More importantly, compared with some cathodes unfavorable for charging reactions in Li‐CO_2_ batteries,^8^, ^10^ Ni‐NG shows a charge capacity up to 9791 mA h g^−1^ with a moderate columbic efficiency of 55.6%. Furthermore, the Ni‐NG cathode shows a stable discharge platform of ≈2.82 V at a current density of 100 mA g^−1^, which is close to the theoretical equilibrium voltage of Li‐CO_2_ batteries.^7^, ^8^, ^9^, ^10^ The improvement in the specific capacity and rate capability of Li‐CO_2_ batteries further confirms the advantages of Ni‐NG composites as the electrocatalyst. The plentiful large pores and channels of Ni‐NG provide enough void volume for the deposition of discharge products, and thus result in stable discharge platform and high discharge capacity. For cycling tests, the cells were discharged and charged with a cutoff capacity of 1000 mA h g^−1^ at 100 or 200 mA g^−1^. The cells exhibit excellent performance over 100 cycles with stable discharge (>2.5 V) and charge (<4.2 V) platforms at 100 mA g^−1^ (Figure 2c). Even at a higher current of 200 mA g^−1^, the batteries could still present superior discharge (>2.7 V) and charge (<4.4 V) platforms with 18 cycles (Figure 2d). The unique porous structure ensures uniform CO_2_ transport and electrolyte infiltration, and promotes the decomposition of the discharge products and the enhancement of the reversibility. Nevertheless, the voltage dropped particularly fast when the current density became much higher, suggesting that the mass transfer of Li^+^, electrons, and CO_2_ could become the rate‐determining step. Compared with Li‐O_2_ batteries, the improvement of rate capability is more critical for Li‐CO_2_ batteries.

**Figure 2:**
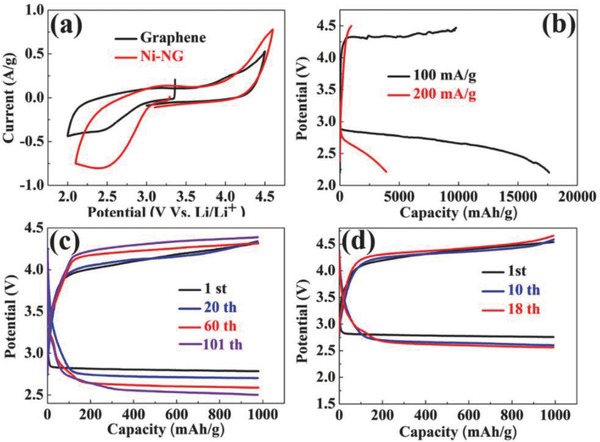
a) CV curves of Li‐CO_2_ batteries with graphene or Ni‐NG cathodes at a scan rate of 0.2 mV s^−1^. b) Discharge–charge profiles of Ni‐NG cathodes at various current densities of 100 and 200 mA g^−1^. Curtailing capacity of 1000 mA h g^−1^ at a current density of c) 100 or d) 200 mA g^−1^.

For the discharged products in the first cycle, X‐ray diffraction (XRD) clearly indicates the formation of the main discharged product of Li_2_CO_3_, and its decomposition after the subsequent charge process (**Figure** **3**a). Besides, the XRD pattern becomes flat and smooth after the charge process. In order to further understand the rechargeability of Li‐CO_2_ cells, differential electrochemical mass spectrometry (DEMS) was utilized to monitor the gas fluent through a Li‐CO_2_ cell during discharge and charge processes. Figure 3b shows the cell voltage and online CO_2_ consumption profile during the discharge process. Here, note that the discharge platform is much lower than that in the above electrochemical tests (Figure 2), since the current density utilized in DEMS tests was ten times larger due to the detection limit of the current DEMS technology. Correspondingly, the charge platform also rises (Figure 3c). Combined with no change of the electrolyte in the nuclear magnetic resonance (NMR) measurements (Figure S6, Supporting Information),^24^, ^25^ the gas evolution profiles during the charging process of Li‐CO_2_ cells suggest the reversible consumption and evolution of CO_2_. In addition, the discharge–charge products were analyzed by Fourier‐transform infrared (FTIR) spectroscopy (Figure S7, Supporting Information). We also used electrochemical impedance spectroscopy (EIS) to characterize the surface states of the electrodes after the first discharge and charge process. The impedance increases apparently after discharge due to the generation of the electrically insulating discharge products, and recovers after the charge process (Figure S8, Supporting Information), indicating the excellent electrochemical activity of Ni‐NG. These results confirm the superiority of Ni‐NG cathodes in terms of the reversibility and stability in Li‐CO_2_ batteries.

**Figure 3:**
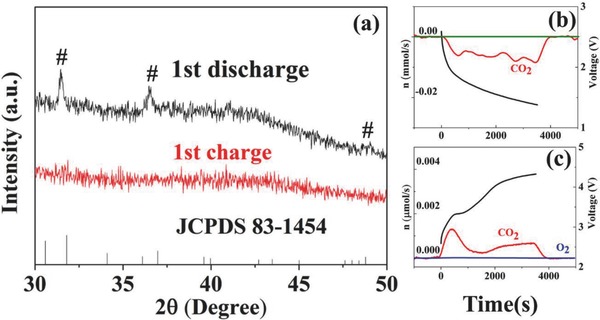
a) XRD patterns after the first discharge and charge. The signal of Li_2_CO_3_ is marked as #. DEMS of b) discharging and c) charging a Li‐CO_2_ battery with Ni‐NG.

Although both the formation and decomposition of Li_2_CO_3_ and the consumption and evolution of CO_2_ during the discharge and charge processes were confirmed, the track of the discharge product of C was still a mystery. The direct observation of the discharge product C in working air cathodes is extremely necessary to understand the total electrochemical reactions of Li‐CO_2_ batteries. First, it is not clear whether the reaction mechanism is the same for Li‐CO_2_ batteries with noble metal or carbon cathodes. Second, different cathodes showed diverse morphologies of amorphous C. Li‐CO_2_ batteries with porous gold as air cathodes exhibited the formation of rough and thick amorphous C; in contrast, thin and massive amorphous C generated in platinum net cathodes.^7^, ^9^ The morphology and distribution of discharge products are closely associated with the reaction process.

To further elucidate the discharge and charge processes of Li‐CO_2_ batteries with Ni‐NG cathodes, the morphologies of discharged and charged cathodes were observed through SEM and TEM (**Figure** **4**). The characterization of charge–discharge processes was limited to the cutoff capacity of 1000 mA h g^−1^ at 100 mA g^−1^. During the first discharge process, the Ni‐NG composites gradually become thickened and high‐density particles densely aggregate on the large space between Ni‐NG (Figure 4a,b). Particularly, different from the fully dense structure of the cathodes after the discharge process for some Li‐O_2_ and Li‐CO_2_ batteries,^7^, ^8^, ^9^, ^26^, ^27^, ^28^ Ni‐NG still maintains the porous structure with wrinkled nanosheets.^29^, ^30^, ^31^ In addition, it is obvious that the discharged products are composed of thin films and agglomerate particles around Ni granules with much more porous and thinner structure than individual graphene,^7^ and Ni‐NG could efficiently contribute to the homogeneous distribution of discharge products. Besides, these agglomerate particles do not belong to enlarged Ni granules, which can also be confirmed by high dispersion of Ni element mapping after the discharging process (Figure S9, Supporting Information). More detailed analyses of the discharge products were achieved through SAED and TEM. In SAED, the morphology of Ni‐NG becomes thickened to divide into different regions after the first discharge process; one is the agglomerated particles which have gathered together (A) and the other is the film covering Ni‐NG (B), which are also observed in SEM. Next, we used SAED to detect the two regions separately. First, Region A (Figure 4c,e) shows more polycrystallization rings than individual Ni‐NG indicating the generation of new phases. Except for the crystal planes of graphene and Ni, other diffraction rings can be confirmed as (−311), (−204), and (311) lattice planes of Li_2_CO_3_ (Figure 4f). Second, Region B (Figure 4d) exhibits almost no rings like some layers of amorphous film completely covering Ni‐NG. Some Li_2_CO_3_ around Ni granules may also be covered by these amorphous films. By combining the NMR result (Figure S6, Supporting Information), we believe that these thin films can be attributed to the newly formed amorphous carbon. Therefore, our Li‐CO_2_ batteries with Ni‐NG cathodes could indeed generate Li_2_CO_3_ and amorphous C according to the reaction of 4Li + 3CO_2_ → 2Li_2_CO_3_+ C.^7^, ^8^, ^9^, ^10^ Through the TEM screening, amorphous C covers the Ni‐NG cathode like a film, and Li_2_CO_3_ prefers to gather together into particles around Ni. More importantly, the unique highly dispersed porous structure with Li_2_CO_3_ particles and large film‐like amorphous C ensures uniform electrolyte distribution around the discharge products, and then enhances the decomposition of the products during the charge and results in an improvement in the reversibility.^31^, ^32^, ^33^

**Figure 4:**
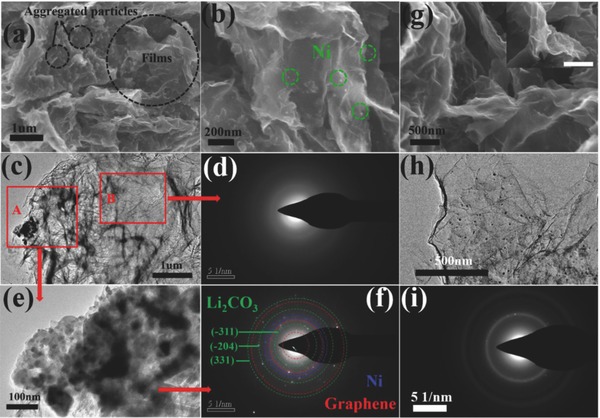
a,b) SEM images with different magnifications of Ni‐NG in the first discharge process. c–f) SAED patterns of Ni‐NG in different regions after the discharge process. g) SEM, h) TEM, and i) SAED of Ni‐NG after the charge process.

After the charge process, the discharge products obviously disappear and the cathode recovers to the porous structure with well‐distributed elements (Figure 4g and Figure S10, Supporting Information). As shown in Figure 4h, Ni‐NG recovers its typical morphology of graphene thin sheets with anchored Ni nanoparticles, while SAED also shows tidy Ni and graphene patterns with part of Li_2_CO_3_ (Figure 4i). As revealed by high‐resolution narrow X‐ray photoelectron spectroscopy (XPS) of Li1s, the peak of Li_2_CO_3_ at 55.4 eV decreases obviously after the subsequent charge process, and only the residual signal can still be distinguished (Figure S11, Supporting Information), consistent with SAED. Even so, the superior cyclic stability indicates that massive active sites on Ni‐NG could contribute to the homogenous distribution of Li_2_CO_3_ and C, and are beneficial to retain the porous structure after electrochemical processes.^27^, ^32^

First‐principles computations were further performed to confirm the superiority of Ni‐NG in Li‐CO_2_ batteries, especially for the catalysis of captured reactants (CO_2_ and Li). The interactions between the reactants and Ni surfaces including (111) and (200) were computed, and the model is shown in Figure S12 (Supporting Information). The interaction between Li and Ni surface is very strong (−0.99 eV for Li/Ni(111) and −1.19 eV for Li/Ni(200)), suggesting that the Ni surface can effectively adsorb Li. For the adsorption of CO_2_, after optimization, the CO_2_ molecule becomes bent due to the strong interaction between CO_2_ and Ni surface. The adsorption energy is −0.36 eV for CO_2_/Ni(111) and −0.54 eV for CO_2_/Ni(200). These results indicate that Ni‐NG can effectively capture the reactants of Li and CO_2_, and demonstrate that Ni particles are active centers for the reaction between Li and CO_2_ in Li‐CO_2_ batteries.

In conclusion, we fabricated a composite of Ni nanoparticles highly dispersed on N‐doped graphene with 3D porous structure. Li‐CO_2_ batteries with Ni‐NG cathodes delivered high discharge capacity and excellent cyclic stability with stable discharge and charge platforms. Meanwhile, the reversible consumption and evolution of CO_2_ were confirmed by DEMS. More importantly, the unique morphology of Ni‐NG cathodes enabled SEM, TEM, and SAED observations of the morphological evolution of the discharge products of agglomerated Li_2_CO_3_ particles and carbon thin films. Therefore, the reversibility of the electrochemical reaction could well be understood in Li‐CO_2_ batteries, and this work would inspire more strategies for developing highly efficient cathodes for rechargeable Li‐CO_2_ batteries.

## Experimental Section

*Materials Preparation*: All the reagents were analytical grade without further purification. Few‐layered graphene was purchased from J&K. In a typical synthesis process, 0.349 g Ni(CH_3_COO)_2_∙4H_2_O, 17.5 mg graphene, and 1.394 g urea were mixed with 35 mL ethylene glycol (EG) through intense agitation for 0.5 h, and ultrasonically treated for 0.5 h homogeneously. Next, the mixed solution was transferred into 50 mL Teflon‐lined stainless steel autoclave and kept at 200 °C for 1 h. After cooling down, the mixture was washed with ethanol for six times, and dried at 80 °C in a vacuum oven. The Ni‐NG composite was obtained by annealing the above mixture at 700 °C in an Ar atmosphere for 10 h.

*Materials Characterization*: XRD was performed on a D/MAX III diffractometer with Cu Kα radiation. Field emission SEM (FESEM) images were obtained on a JEOL‐JSM7500 microscope. TEM and HRTEM images were taken on FEITecnai G2F‐20, and XPS was performed on Axis Ultra DLD (Kratos Analytical). FTIR spectroscopy was conducted on NicoletMAGNA‐560 FTIR spectrometer by using KBr pellets, and Raman spectra on a Renishaw inVia Raman spectrometer equipped with a 632.8 nm laser. TG‐DTA was performed on Rigaku PTC‐10 A TG‐DTA analyzer, and Vario EL CUBE elemental analyzer was used to quantitatively confirm the contents of C and N. ^1^H NMR was recorded on a Bruker AV400 spectrometer. DEMS was measured through a commercial quadrupole mass spectrometer (Hiden Analytical) and a home‐made Swagelok‐type DEMS cell with two PEEK capillary tubes as purge gas inlet and outlet. The details can be found in a previous report.^34^

*Electrochemical Tests*: The electrochemical behaviors were measured in Swagelok cells with a 1.0 cm^2^ hole placed on the cathode which enabled CO_2_ to flow in. The cells were assembled in a glove box filled with high‐purity argon (O_2_ and H_2_O < 0.1 ppm). For the cathode preparation, a slurry, obtained by mixing Ni‐NG and polyvinylidenefluoride (PVDF) with the mass ratio of 9:1, was uniformly deposited on a circular piece of carbon paper (12 mm in diameter; mass loading of 0.3–0.5 mg), and then dried in an oven at 80 °C. Li foil (14 mm) was used as the anode, and polytetrafluoroethylene (PTFE) membrane (18 mm) as the separator. The electrolyte was 1 mol L^−1^ lithium bis(trifluoromethanesulfonyl)imide (LiTFSI) dissolved in tetraethylene glycol dimethyl ether (TEGDME). Discharge/charge tests were conducted on LAND‐CT2001A testers, and the cells were discharged to 2.2 V and then recharged to 4.5 V. CV and EIS were conducted on a Zahner‐Elektrik IM6e electrochemical workstation within 2–4.5 V. Cyclic tests were controlled with the cutoff capacity of 1000 mA h g^−1^ at a current density of 100 and 200 mA g^−1^, that is, the cells were discharged and charged for 10 and 5 h, respectively.

*Computational Methods*: First‐principles computations on basis of density functional theory (DFT) were performed with a plane‐wave technique as implemented in the Vienna Ab initio simulation package (VASP).^35^ Projector augmented plane wave (PAW) pseudopotential was applied to describe ion–electron interactions.^36^ A 550 eV cutoff energy for the plane‐wave basis set was adopted in all computations. The generalized gradient approximation (GGA) involving Perdew, Burke, and Ernzerhof (PBE) was used for calculating the exchange‐correlation energy.^37^ The Brillouin zone was represented by Monkhorst–Pack special *k*‐point meshes of 11 × 5 × 1 for Ni(111) and 11 × 5 × 1 for Ni(200). The model is shown in Figure S12 (Supporting Information). Considering weak interactions, the DFT‐D3 method with Beck–Jonson damping was adopted.^38^, ^39^ To avoid any artificial interactions between the periodically repeated images along *c*‐axis, a vacuum space with ≈20 Å was inserted. In the computations, the lower three layer atoms were fixed and other atoms were fully relaxed. The adsorption energy (*E*_ads_) of adsorbates was calculated from the equation *E*_ads_ = *E*_tot_ − *E*_M_ − *E*_N_, where *E*_tot_, *E*_M_, and *E*_N_ represent the total energy of the complex of the Ni substrate and adsorbate, the isolated atom/molecule, and the Ni substrate, respectively.

## Conflict of Interest

The authors declare no conflict of interest.

## Supporting information

Supplementary
