# Supplementary material for: Verifying the Rechargeability of Li‐CO2 Batteries on Working Cathodes of Ni Nanoparticles Highly Dispersed on N‐Doped Graphene
Source: Adv Sci (Weinh). 2017 Nov 10;5(2):1700567. doi: 10.1002/advs.201700567 (PMC5827515; doi:10.1002/advs.201700567)
Supplement: Supplementary file 1 — Supplementary [file ADVS-5-1700567-s001.pdf]

Copyright WILEY-VCH Verlag GmbH & Co. KGaA, 69469 Weinheim, Germany, 2017.

Supporting Information

for *Adv. Sci.,* DOI: 10.1002/advs.201700567

Verifying the Rechargeability of Li-CO_2 Batteries on Working Cathodes of Ni Nanoparticles Highly Dispersed on N-Doped Graphene

Zhang Zhang, Xin-Gai Wang, Xu Zhang, Zhaojun Xie, Ya-Nan Chen, Lipo Ma, Zhangquan Peng, and Zhen Zhou**

Supplementary Materials

Verifying the rechargeability of Li-CO_2 batteries on working cathodes of Ni nanoparticles highly dispersed on N-doped graphene

Zhang Zhang,[#] Xin-Gai Wang,[#] Xu Zhang, Zhaojun Xie, Ya-Nan Chen, Lipo Ma, Zhangquan Peng, Zhen Zhou**

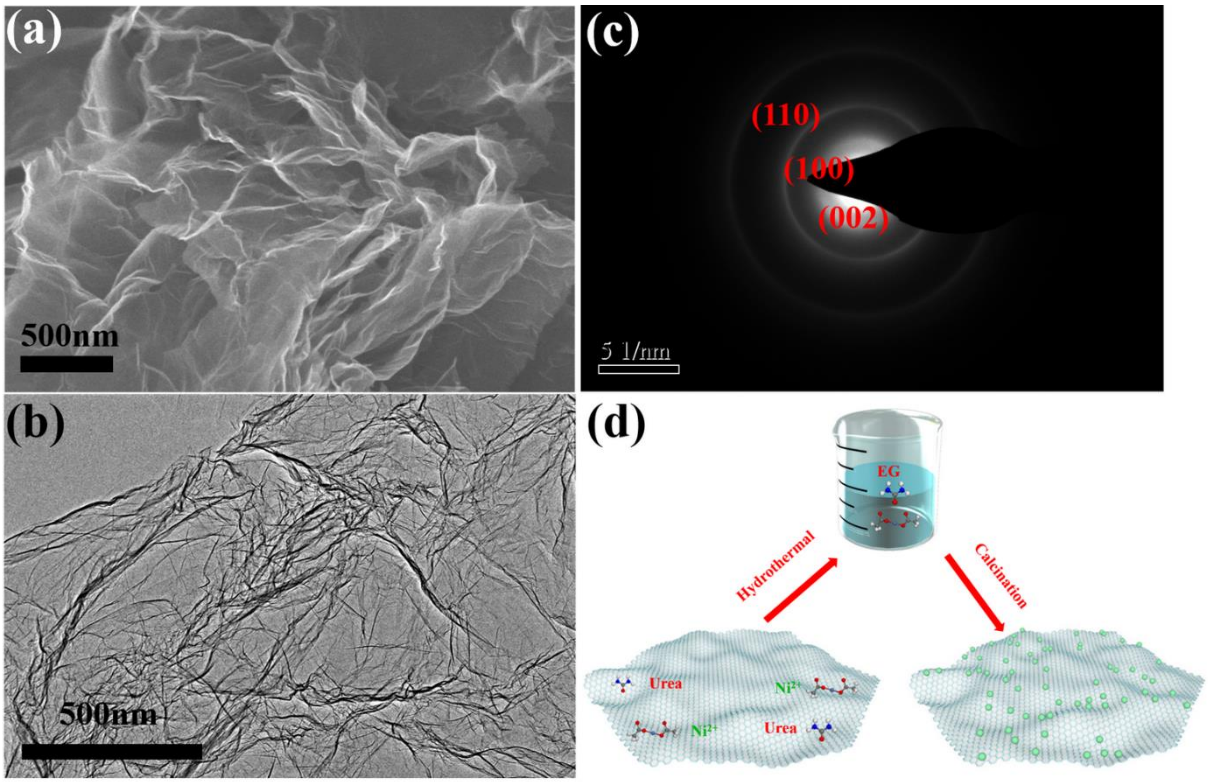

Figure S1. (a) SEM, (b) TEM, and (c) SAED of graphene. (d) Schematic illustration of Ni-NG preparation.

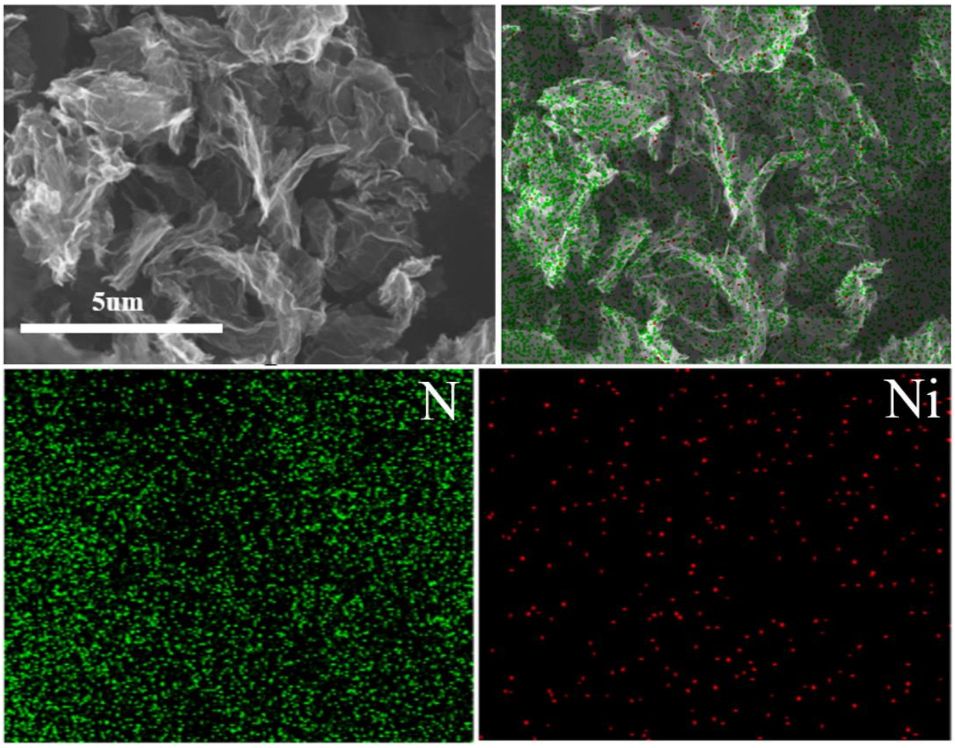

Figure S2. Element mapping images of Ni-NG.

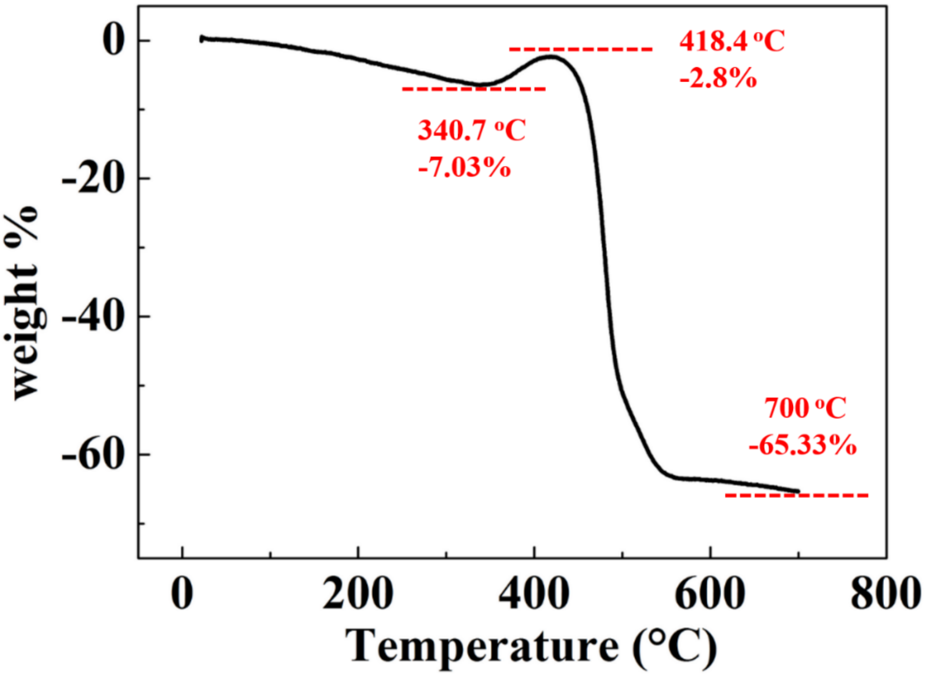

Figure S3. TG-DTA curve of Ni-NG.

The weight loss can be mainly ascribed to the slow decomposition (before 340 °C) along with the oxidation of Ni and dramatic oxidation of graphene. Thus, the Ni content is estimated to be ~27.2wt%.

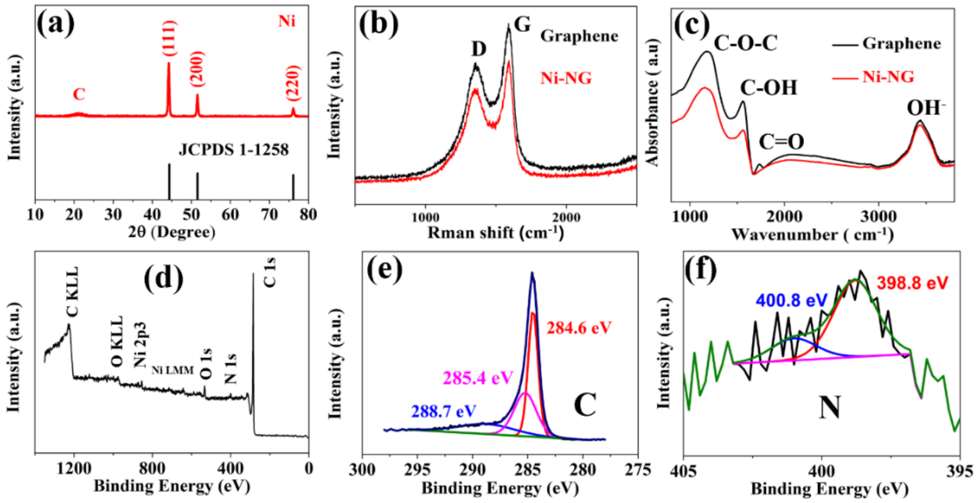

Figure S4. (a) XRD patterns, (b) Raman, and (c) FTIR spectra of Ni-NG composite. XPS survey spectrum (d), (e) C1s and (f) N1s.

Figure S4a shows the XRD patterns of Ni-NG; aside from the peak of graphene, three peaks are well assigned to the (111), (200), and (220) planes of cubic Ni (JCPDS 1-1258).[1] In order to accurately detect the change of carbon structure, we utilized Raman and FTIR to test graphene and Ni-NG. The Raman spectra of graphene and Ni-NG contain two typical peaks of G and D bands (Figure S4b). Briefly, the G band represents the characteristic of graphitic layers and relates to the stretching vibration of C-C bonds, while the D band indicates the disorder induced feature.[2,3]. Compared with graphene ($I_D/I_G \approx 0.77$), Ni-NG shows a slight increase for $I_D/I_G \approx 0.79$ which reveals a little change on carbon structures. This demonstrates that Ni particles are tactfully integrated into graphene structure without impairing the electrical conductivity which is significant for realizing high performances in Li-CO_2 batteries.[2] Besides, the upshifts in ω_G could be used to characterize the number of graphene layers,[4] and the positions of G peak in graphene and Ni-NG are all located in 1589 cm^{-1}, indicating the morphology retention without aggregation after hydrothermal and calcination process. In addition, the FWHM (full width at half maximum) of G peak has obviously been narrowed in Ni-NG, and this proves that N doping has influenced the structure of Ni-NG compared with individual graphene, which was decomposed from urea after the hydrothermal and calcination process.[5].

In FTIR (Figure S4c), except for common peaks between Ni-NG and graphene around 3439 cm^{-1}, 1570 cm^{-1} and 1160 cm^{-1} that could be assigned to OH-, C-OH and C-O-C, respectively, the peak at 1740 cm^{-1} (C=O) obviously decreases in Ni-NG compared with graphene, further decreasing the oxygen content to promote the electronic conductivity. XPS further accurately confirms the electronic structures of C and N. Figure S4d shows the survey spectrum of Ni-NG. In different high-resolution narrow spectra, the typical C-containing groups can be observed in Figure S4e, and the major shark peak at 284.6 eV related to the sp^2 C-sp^2 C indicates that most carbon atoms are arranged in conjugated honeycomb lattices. The peaks at 285.4 and 288.7 eV are assigned to N-sp^2 C or C-O and N-sp^3 C or C=O bonds, respectively.[6-9] The high-resolution N1s peak at 398.8 eV demonstrates the main pyridinic-N type and 400.8 eV can be assigned to pyrrolic-N type (Figure S4f). O and Ni high-resolution narrow spectra are presented in Figure S5. Element analyses quantitatively confirmed the content of N to be 3.95 wt% (Table S1).

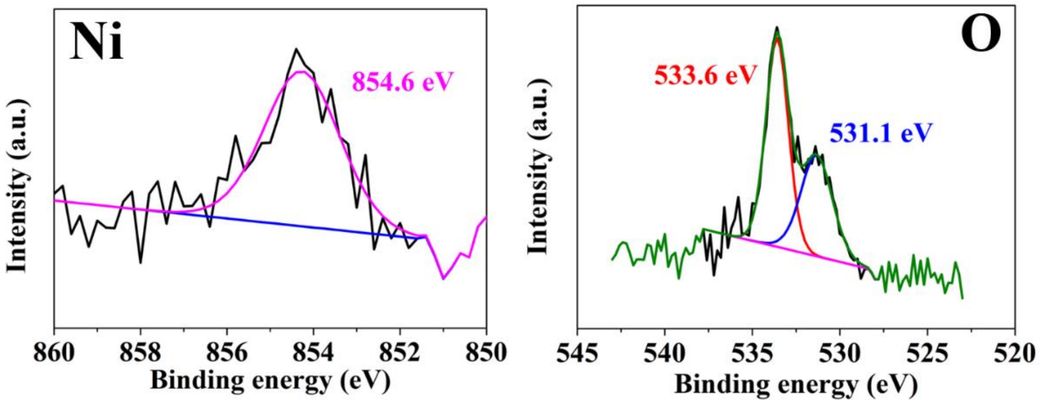

Figure S5. Ni, and O high-resolution XPS narrow spectra.

Table S1. Element analyses of Ni-NG.

Element	Ni	C	N	H
Amount (wt%)	27.2	56.6	3.95	6.56

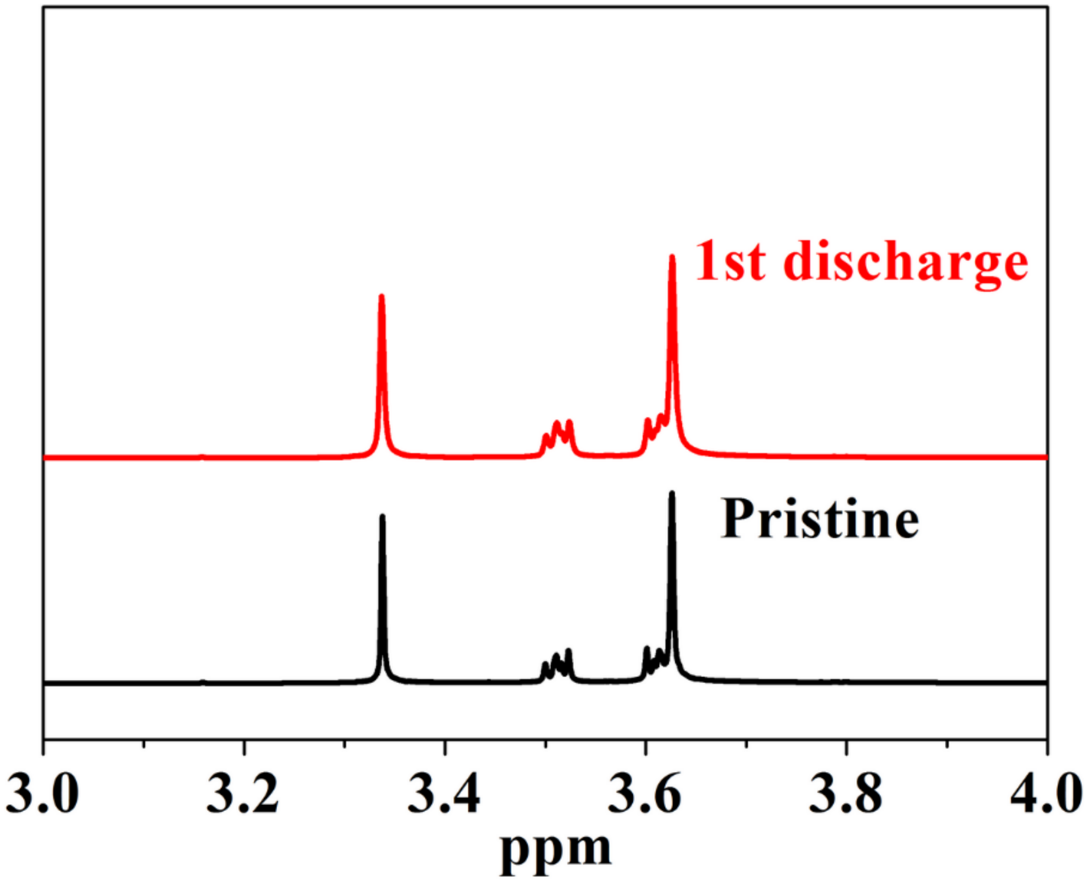

Figure S6. ^{1}H NMR analysis on the pristine electrolyte and the one after discharge.

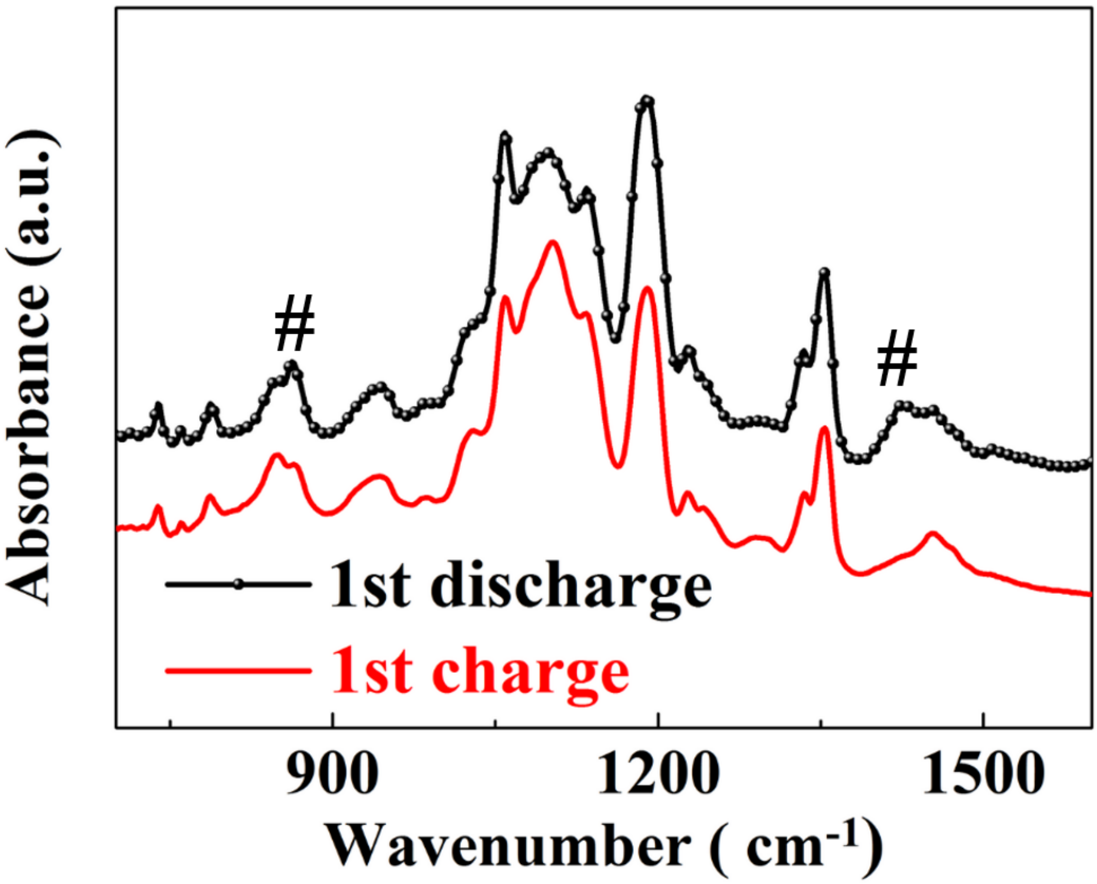

Figure S7. FTIR for the first discharge and charge. The signal of Li_2CO_3 is marked as #

The characteristic peaks of Li_2CO_3 were observed at 1430 cm^{-1} and 865 cm^{-1}, which are consistent with the standard patterns of Li_2CO_3 and previous reports.[10-15] Other peaks might be the electrolyte of lithium bis(trifluoromethanesulfonyl)imide (LiTFSI) in tetraethylene glycol dimethyl ether (TEGDME), which could also be clearly observed in the FTIR spectrum of the charged cathode. After the charge process, Li_2CO_3 disappears obviously.

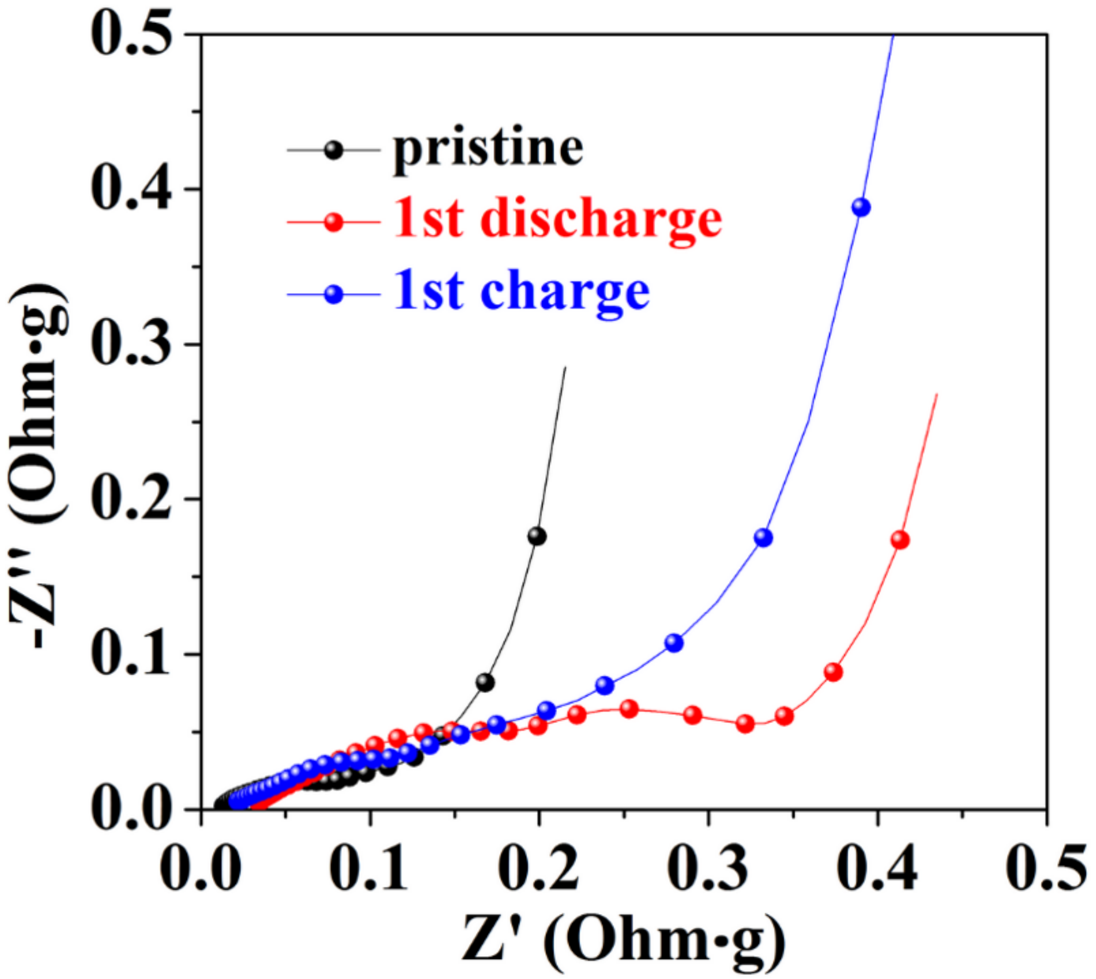

Figure S8. EIS for the cells assembled with Ni-NG cathodes, as well as those after the first discharge and the subsequent charge.

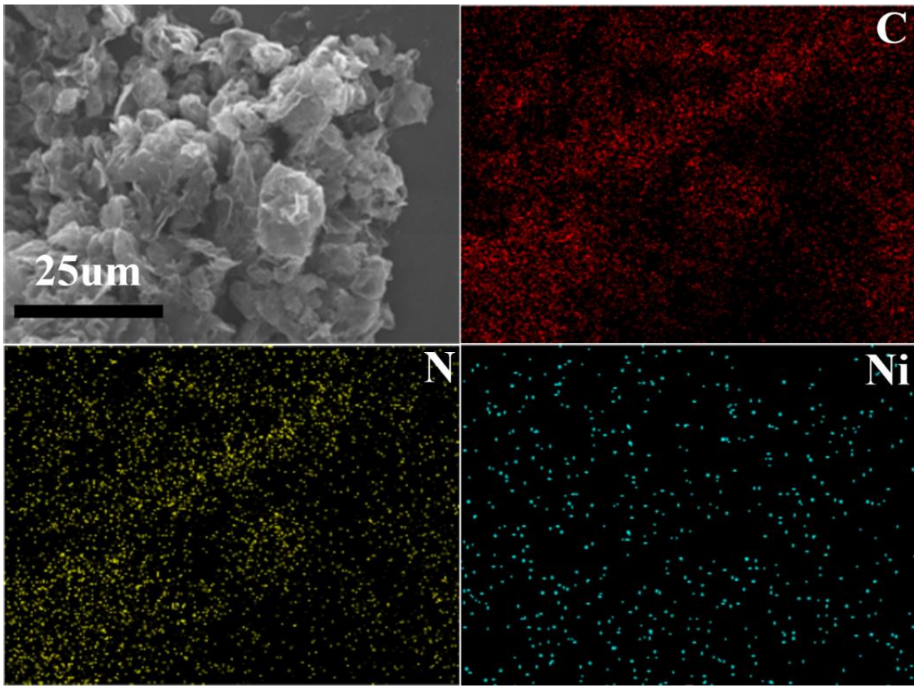

Figure S9. Element mapping images of SEM after the discharge process.

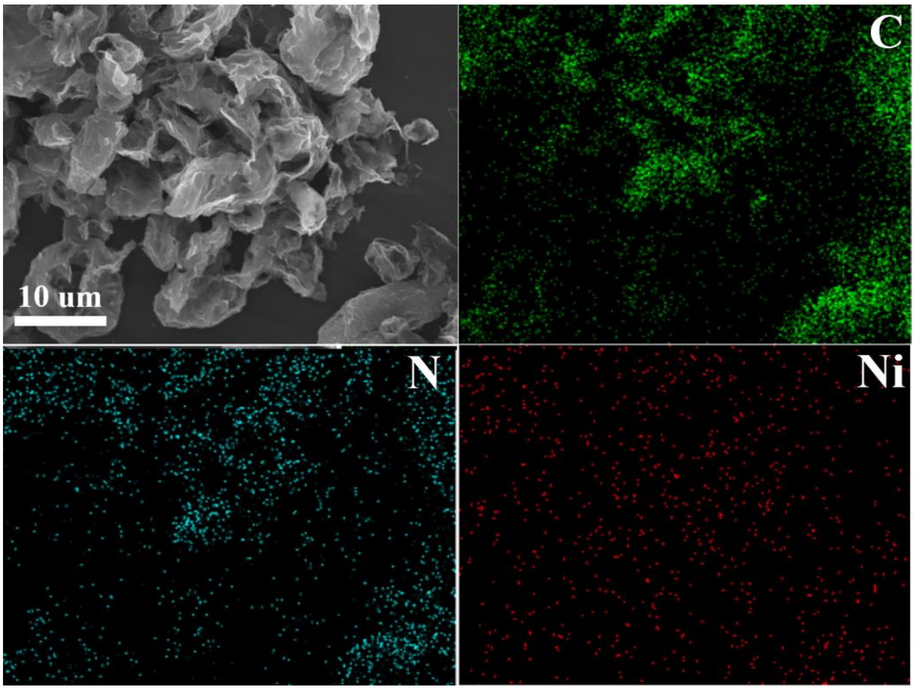

Figure S10. Element mapping images of SEM after the charge process.

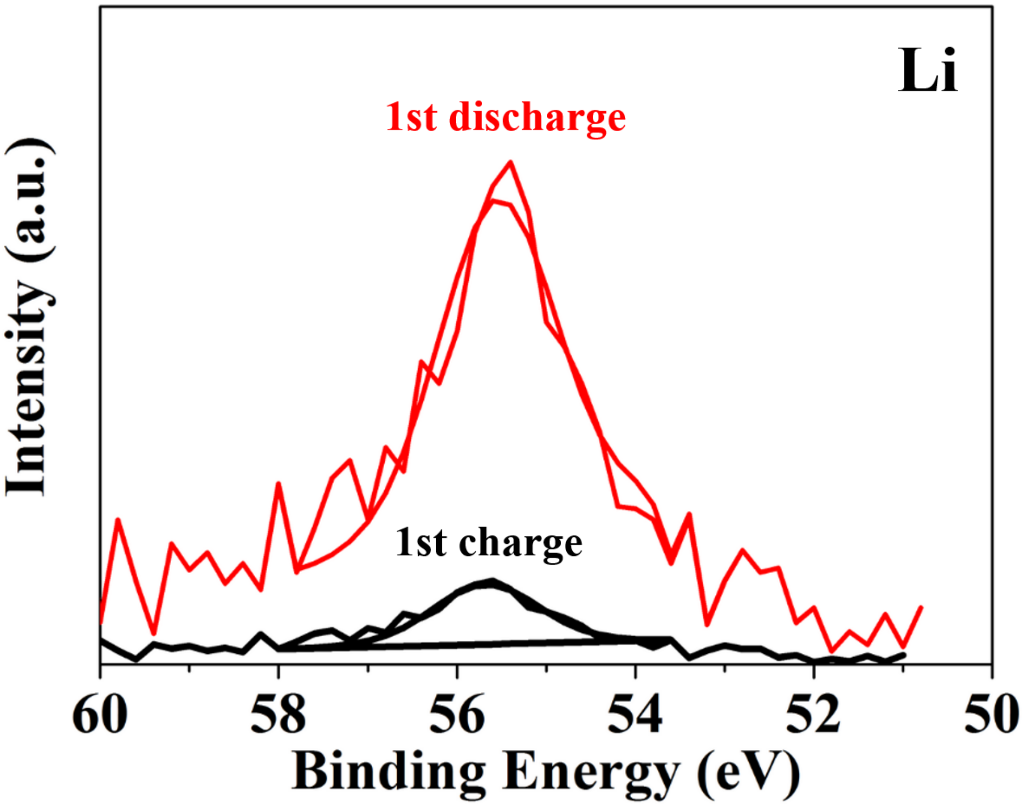

Figure S11. Li1s XPS of the cathode after discharge-charge processes.

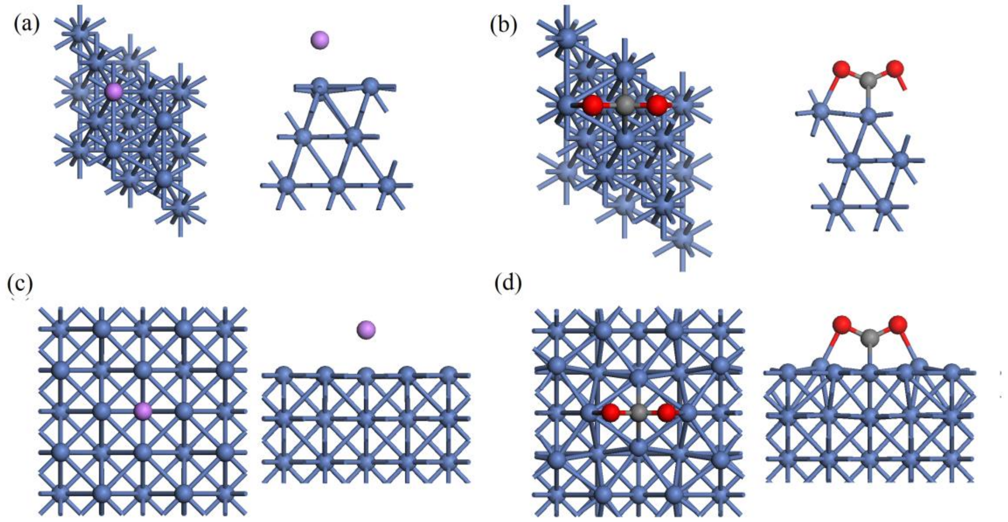

Figure S12 Top (left) and sight (right) view of the optimized energetically most favorable structures of (a,c) Li and (b,d) CO_2 adsorbed on Ni(111) and (200) surfaces, respectively. The blue, purple, grey and red balls represent Ni, Li, C and O, respectively.

References

[1] Z. Zhang, L. Su, M. Yang, M. Hu, J. Bao, J. Wei, Z. Zhou, *Chem. Commun.* **2014**, *50*, 776.

[2] Y. Zhong, M. Yang, X. Zhou, J. Wei, Z. Zhou, *Part. Part. Syst. Charact.* **2015**, *32*, 104.

[3] A. C. Ferrari, D. M. Basko, *Nat. Nanotechnol.* **2013**, *8*, 235.

[4] M. S. Dresselhaus, G. Dresselhaus, M. Hofmann, *Phil. Trans. R. Soc. A* **2008**, *366*, 231.

[5] S. Berciaud, S. Ryu, L. E. Brus, T. F. Heinz, *Nano Lett.* **2009**, *9*, 346.

[6] Z. H. Sheng, L. Shao, J. J. Chen, W. J. Bao, F. B. Wang, X. H. Xia, *ACS nano* **2011**, *5*, 4350.

[7] Z. Zhang, J. Bao, C. He, Y. Chen, J. Wei, Z. Zhou, *Adv. Funct. Mater.* **2014**, *24*, 6826.

[8] Z. Zhang, Y. Chen, J. Bao, Z. Xie, J. Wei, Z. Zhou, *Part. Part. Syst. Charact.* **2015**, *32*, 680.

[9] M. Yang, Y. Zhong, J. Bao, X. Zhou, J. Wei, Z. Zhou, *J. Mater. Chem. A* **2015**, *3*, 11387.

[10] Z. Zhang, Q. Zhang, Y. Chen, J. Bao, X. Zhou, Z. Xie, J. Wei, Z. Zhou, *Angew. Chem. Int. Ed.* **2015**, *54*, 6550.

[11] X. Zhang, Q. Zhang, Z. Zhang, Y. Chen, Z. Xie, J. Wei, Z. Zhou, *Chem. Commun.* **2015**, *51*, 14636.

[12] J. J. Xu, Z. L. Wang, D. Xu, L. L. Zhang, X. B. Zhang, *Nat. Commun.* **2013**, *4*, 2438.

[13] S. A. Freunberger, Y. Chen, N. E. Drewett, L. J. Hardwick, F. Barde, P. G. Bruce, *Angew. Chem. Int. Ed.* **2011**, *50*, 8609.

[14] S. Xu, S. K. Das, L. A. Archer, *RSC Adv.* **2013**, *3*, 6656.

[15] Y. Liu, R. Wang, Y. Lyu, H. Li, L. Chen, *Energy Environ. Sci.* **2014**, *7*, 677.
